# Artificial Intelligence-Integrated Proteome-Wide Target Prioritization for Drug Discovery against Multidrug-Resistant *Neisseria gonorrhoeae*

**DOI:** 10.1021/acsinfecdis.6c00521

**Published:** 2026-09-09

**Authors:** Ravi Kant, Saurabh Mazumdar, Ananya Mahajan, Myron Christodoulides

**Affiliations:** † Computational Drug and Vaccine Discovery Laboratory, Faculty of Applied Sciences and Biotechnology, 375605 Shoolini University, Solan 173229, Himachal Pradesh, India; ‡ Neisseria Research Group, Molecular Microbiology, School of Clinical and Experimental Sciences, University of Southampton Faculty of Medicine, Southampton General Hospital, SO16 6YD Southampton, U.K.

**Keywords:** *Neisseria gonorrhoeae*, antimicrobial resistance (AMR), proteome-wide target fishing, core genome analysis, essential genes, AI drug discovery

## Abstract

*Neisseria gonorrhoeae* causes ∼82.4 million infections annually and is rapidly approaching an untreatable status. The pathogen has developed resistance to every antibiotic class introduced since the sulfonamide era. Ceftriaxone, the last WHO- and CDC-recommended empirical therapy, is now compromised by high-level resistance in extensively drug-resistant lineages reported across multiple continents. No licensed vaccine exists, and no antibiotic with a novel mechanism has been introduced for this pathogen in over 40 years. Conventional drug discovery is too slow and insufficiently resistance-aware to address this escalating threat. In this review, we examine in silico proteome-wide target fishing as a rapid, systematic approach. The approach applies six biologically informed filters to the *N. gonorrhoeae* core proteome: core-genome definition, host-homology exclusion, essentiality screening, metabolic chokepoint identification, subcellular localization, and resistance-aware prioritization. Across data sets spanning 12–69 clinical strains, this pipeline reduces 2000–12,300 protein-coding sequences to 12–30 high-confidence targets. An integrated artificial intelligence (AI)/machine learning (ML) scoring layer further ranks candidates using sequence features, AlphaFold2 structures, protein–protein interaction networks, and genotype–phenotype data from ∼20,000 clinical isolates. Five targets showed consistent cross-study prioritization: LpxC, MurA, FabI, DapD, and NGFG_RS03485. Of these, only LpxC has experimental inhibitor validation against multidrug-resistant gonococci; the remaining candidates are supported computationally but lack biochemical confirmation. Bridging this validation gap remains the critical bottleneck. However, advances in pan-genome analysis, structural prediction, and generative AI for compound design provide a more tractable route to novel therapeutics than previously available.

## Graphical abstract

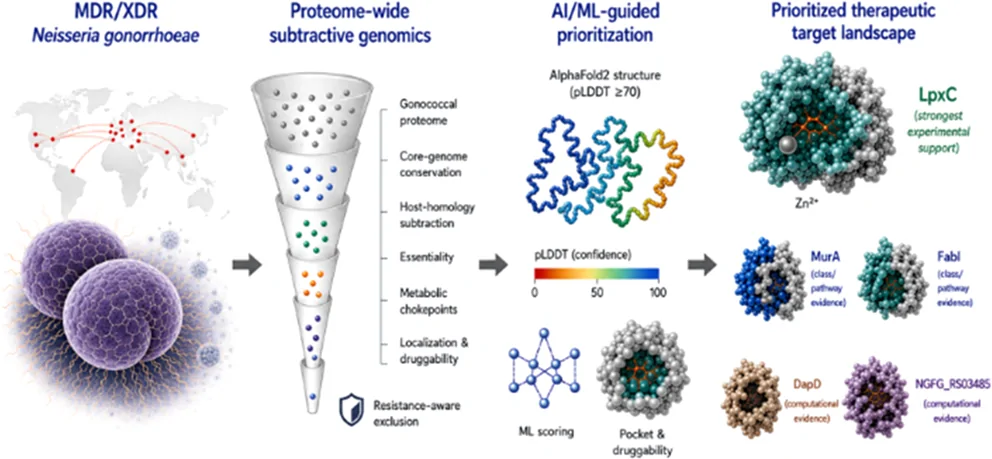

## Introduction

### The Burden of Gonorrhea and Antimicrobial Resistance (AMR)

Gonorrhea, caused by *Neisseria gonorrhoeae* (gonococcus) is the second most prevalent bacterial sexually transmitted infection worldwide and one of the most consequential for reproductive health outcomes. The World Health Organization (WHO) estimates approximately 82–100 million new cases annually among adults aged 15–49 years worldwide, reflecting the persistent and substantial global burden of gonorrhea.^1,2^ This burden is highest across the WHO African and Western Pacific regions, but gonorrhea remains endemic globally, with over 1.5 million annual cases in the United States and upward trends documented across Europe, South Asia, and Southeast Asia.^2^ Gonococci infect the mucosal epithelium of the lower genital tract, and uncomplicated infection in men is characterized by a purulent urethritis and pain, and in women by a purulent ecto/endocervicitis. The global burden of gonorrhea is probably under-reported due to the high incidence of asymptomatic infection. In women with asymptomatic infection, there is a possibility that gonococci ascend into the upper reproductive tract, and the host response to ascending infection can be pelvic inflammatory disease, ectopic pregnancy, endometritis and salpingitis (inflammatory responses in the endometrium and Fallopian tubes), tubal infertility, and augmented HIV transmission.^3,4^ The clinical burden is compounded in low and middle income countries where diagnostic infrastructure is limited.^1^

*N. gonorrhoeae* has acquired resistance to every therapeutic class used against it since the sulfonamide era, and penicillins, tetracyclines, spectinomycin, fluoroquinolones, early cephalosporins, and azithromycin have each been successively rendered unreliable.^5^ A single intramuscular dose of 500 mg of ceftriaxone is currently recommended by the Centers for Disease Control and Prevention (CDC) for treating uncomplicated gonorrhea and it is endorsed by the WHO as the last widely effective empirical therapy. However, its efficacy is now threatened by the emergence of extensively drug-resistant (XDR) gonococcal lineages. For example, the XDR strains H041, isolated in Japan,^6^ and F89, identified in France,^7^ both exhibited high-level ceftriaxone resistance alongside multidrug resistance (MDR) profiles, and similar isolates have since been characterized in many countries.^8–11^ The WHO, Gonococcal Antimicrobial Surveillance Programme (GASP) and Enhanced Gonococcal Antimicrobial Surveillance Programme (EGASP) surveillance platforms continue to document rising rates of reduced ceftriaxone susceptibility across Southeast Asia, the Americas, and Europe.^2^ Consequently, there is an urgent need to develop new therapies that target previously unexploited gonococcal pathways. The opportunity to identify such targets is rapidly narrowing.

### Why Conventional Target Discovery Has Failed for *N. gonorrhoeae*

Decades of sustained pre-clinical and clinical research have produced neither a licensed gonococcal vaccine^12,13^ nor an antibiotic acting through a mechanistically novel target.^14,15^ These are not incidental failures, and they reflect properties of the gonococcus that specifically defeat both immunological and pharmacological approaches. Vaccine development has been frustrated by extensive antigenic and phase variation across the organism’s principal surface structures, i.e., the pilus, lipooligosaccharide (LOS) and several outer membrane (OM) proteins.^12,13^ Natural infection does not establish protective immune memory, and reinfection with the same or different strains is well documented.^16^ Without an immunological scaffold analogous to that exploited in pneumococcal or meningococcal vaccine programs, rational antigen selection remains unresolved.

Drug discovery faces a different set of obstacles. *N. gonorrhoeae* is an obligate human pathogen requiring CO_2_-enriched environments, specific supplements, and precise cultivation conditions that complicate high-throughput experimental work. Constructing saturating transposon insertion libraries for genome-wide essential gene mapping is substantially more demanding in this organism than in well-characterized bacteria such as *Escherichia coli* or *Staphylococcus aureus*, resulting in comparatively limited gonococcal essentiality data sets.^14,17^ Consequently, many computational pipelines rely partly on cross-species inference, introducing uncertainty into early-stage target identification. The rapid emergence of AMR deepens this problem further. Even recently introduced agents such as Zoliflodacin and Gepotidacin,^18^ developed specifically to treat MDR gonococcal infections, are already under close surveillance for resistance-associated mutations and reduced susceptibility in clinical isolates,^19,20^ highlighting the organism’s exceptional evolutionary adaptability.

### The In Silico Proteome-to-Target Paradigm

In antibacterial drug discovery for treating human infections, a “target” refers to a bacterial protein, enzyme, or biological pathway whose inhibition disrupts pathogen survival, replication, or virulence, with the targeting drug itself showing an acceptable safety profile for humans. Identifying such targets experimentally in *N. gonorrhoeae* has proven particularly difficult because of the organism’s fastidious growth requirements, high genetic variability, limited gonococcal essentiality data sets, and rapid acquisition of AMR. Computational target fishing emerged as a strategy to address these limitations.^14,21^ The approach systematically interrogates the gonococcal proteome through sequential biological filters — including essentiality, host nonhomology, metabolic indispensability, subcellular localization, and structural druggability — to generate a prioritized shortlist of proteins suitable for downstream experimental validation.^14,21,22^ Earlier subtractive genomics studies used the ∼2000-protein *N. gonorrhoeae* FA1090 reference strain proteome as input, whereas more recent analyses incorporated core proteomes derived from 12–69 clinical *N. gonorrhoeae* genomes, expanding the searchable sequence space to >12,000 proteins before sequential filtering. Across these data sets, the pipeline typically narrows the candidate pool to approximately 12–30 high-priority targets.

Where contemporary implementations depart from their predecessors is in the addition of machine learning (ML) and artificial intelligence (AI) as a second analytical layer above these binary filters.^17^ Earlier pipelines returned unranked lists of candidates that passed sequential criteria; ML-based druggability models trained on large annotated data sets assign each candidate a continuous probability score integrating sequence features, predicted structural properties, protein–protein interaction network topologies, and pharmacological precedents.^17^ This converts the pipeline output from a simple filtered list into a prioritized ranking of candidate targets. More recent pipelines also incorporate resistance-aware exclusion, using large-scale gonococcal genome surveillance data to identify proteins whose encoding genes already harbor resistance-associated mutations in circulating clinical isolates, a feature absent from earlier pre-2020 computational studies.^17,21^ Together, these advances provide a more precise framework for gonococcal drug target identification than earlier approaches based solely on laboratory gene disruption studies, essentiality mapping, or conventional computational analyses.^21,22^

### Scope, Objectives, and Search Strategy

In this review, we provide a systematically structured and critically evaluative account of the in silico target fishing pipeline applied to the *N. gonorrhoeae* proteome. It addresses three specific objectives: (i) the six-step subtractive genomics pipeline, including resistance-aware exclusion as a post-2020 methodological addition; (ii) the integration of ML druggability scoring, AMR genotype–phenotype prediction, deep learning structure prediction, and generative AI compound design; and (iii) a consolidated and critically appraised summary of the highest-priority druggable target candidates, with explicit distinction between computational prediction and experimental validation.

Literature was identified through PubMed/MEDLINE and Scopus using the search strings (″*Neisseria gonorrhoeae*″) AND (″drug target″ OR ″therapeutic target″) AND (″in silico″ OR ″computational″ OR ″subtractive genomics″ OR ″bioinformatics″) and (″target fishing″) AND (″gonorrhoeae″ OR ″Gram-negative″). Secondary research combined (″machine learning″ OR ″artificial intelligence″ OR ″deep learning″) AND (″*Neisseria gonorrhoeae*″ OR ″AMR prediction″). The date range was 2004–2025. Inclusion required original computational identification of target candidates from the *N. gonorrhoeae* proteome or pan-genome data, or ML and AI frameworks with direct application to gonococcal target identification, AMR prediction, or de novo compound design against this organism.

## *N. Gonorrhoeae* Proteome: Genomic Context and Target Selection Rationale

### Genome Architecture, Pan-Genome Structure, and Plasticity

*N. gonorrhoeae* encodes approximately 2000 proteins within a 2.1 Mbp chromosome, and this compact genome sustains one of the highest rates of horizontal gene transfer among obligate bacterial pathogens^21,23^ (Figure 1). The FA 1090 reference strain chromosome carries roughly 2000 copies of the 10-bp DNA uptake sequence 5′-GCCGTCTGAA-3′, enabling selective but highly efficient acquisition of exogenous DNA.^24^ The dominant source of exogenous DNA is commensal *Neisseria* colonizing the nasopharynx, and this interspecies exchange with gonococci that have entered the oral cavity and colonized the nasopharyngeal mucosa, is the primary mechanism by which clinically relevant resistance determinants enter and spread through gonococcal lineages.^24–27^

**Figure 1:**
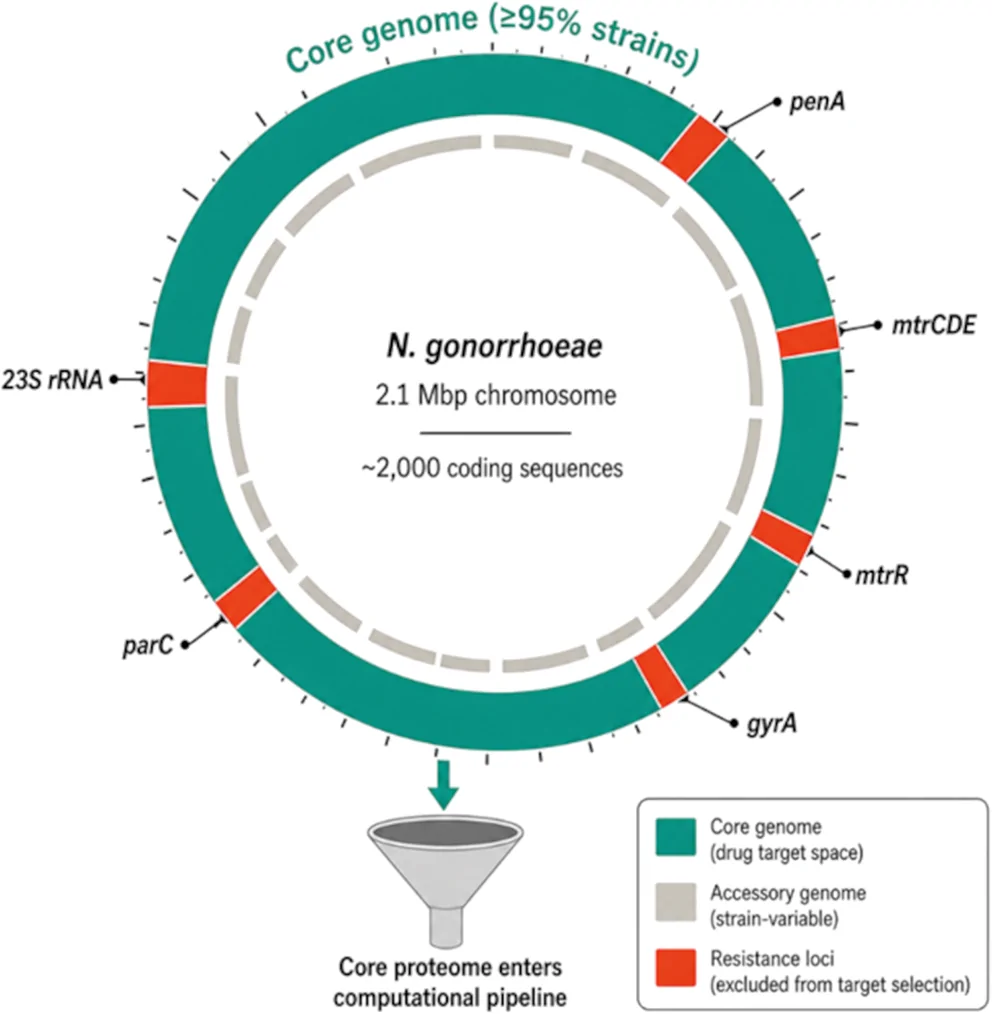
Genome organization and pan-genome structure of *N. gonorrhoeae*. Schematic overview of the gonococcal chromosome highlighting DNA uptake sequences (DUS), horizontal gene transfer, and the open pan-genome organization consisting of conserved core genes and variable accessory genomic elements across clinical strains.

The result is an open pan-genome: species-level coding diversity substantially exceeds what any single isolate encodes, with strains diverging in mobile elements, plasmid content, and accessory gene repertoires. Pan-genome analysis of 69 complete gonococcal genomes by Qasim et al. resolved a conserved core proteome within this highly variable background.^21^ Only proteins encoded by core genes are present across the full range of circulating clinical strains and are therefore valid targets for a broadly effective drug. Compounds that engage proteins absent even from a small proportion of isolates could result in decreased coverage against those gonococci.^21,23^ The multi-genome approach of Qasim et al. is the methodologically sound starting point for any gonococcal target identification study.^21^

### Resistance Determinants That Must Be Excluded from Target Selection

Satisfying classical computational filters is necessary but not sufficient. Several gonococcal proteins score well on essentiality, host nonhomology, and subcellular localization, yet are unsuitable as novel targets because resistance to their inhibition is already established in circulating clinical populations. This was confirmed with the 2023 French XDR isolates F95 and F96: both strains carried convergent resistance mutations across *penA*, *mtrR*, *gyrA*, *parC*, and all four 23S rRNA gene copies, compromising β-lactam, fluoroquinolone, and macrolide targets within a single clinical background.^7^

Penicillin Binding Protein 2 (PBP2), encoded by *penA*, is targeted by ceftriaxone and meets every classical druggability criterion. The mosaic *penA*-60.001 allele, carrying active-site substitutions that impair cephalosporin binding, is now documented in XDR isolates across multiple continents, and PBP2 is categorically excluded.^7^ The MtrCDE efflux pump is upregulated by a well-characterized 1-bp deletion in the *mtrR* promoter and by *mtrR* coding mutations; both mechanisms are prevalent in contemporary isolates and drive resistance to structurally diverse antimicrobials, disqualifying these components as novel targets.^7^ GyrA Ser91/Asp95 and ParC Ser87 quinolone resistance substitutions are near-universally fixed in high-burden regions.^7^ The A2059G mutation, required in all four 23S rRNA chromosomal copies for high-level azithromycin resistance, is documented in clinical isolates and similarly forecloses this target class.^7^ Excluding *penA*, *mtrCDE*/*mtrR*, *gyrA*/*parC*, and 23S rRNA loci as an obligatory upstream filter separates a resistance-informed pipeline from one that reproduces already-compromised candidates, a step omitted by most of the pre-2020 gonococcal computational studies^14,21^ (Figure 1).

## The In Silico Target Fishing Pipeline: Genomics-Based Methods

Identifying druggable proteins computationally from a bacterial proteome demands a carefully constructed, multi-stage filtration workflow in which each successive step eliminates proteins unsuitable for therapeutic targeting on specific, biologically grounded criteria. For *N. gonorrhoeae*, this challenge is sharpened further by a genome marked by genetic plasticity, well-documented horizontal gene transfer with commensal *Neisseria*, and a track record of evolving resistance to every antibiotic class introduced against it. The six-step pipeline reviewed here (Figure 2, Table 1) synthesizes methodological advances reported across four representative gonococcal proteome-wide target identification studies published between 2009 and 2023 from Barh and Kumar,^14^ Tanwer et al.,^28^ Omeershffudin and Kumar^29^ and Qasim et al.^21^ A consolidated list of candidate therapeutic proteins identified across these studies is provided in Table S1. These studies were selected because they are the only studies that have applied systematic subtractive genomics or pan-genome–based computational workflows to prioritize therapeutic targets in *N. gonorrhoeae* and collectively illustrate the progression from early essentiality filtering approaches to more recent resistance-aware and multi-strain analytical frameworks.

**Figure 2:**
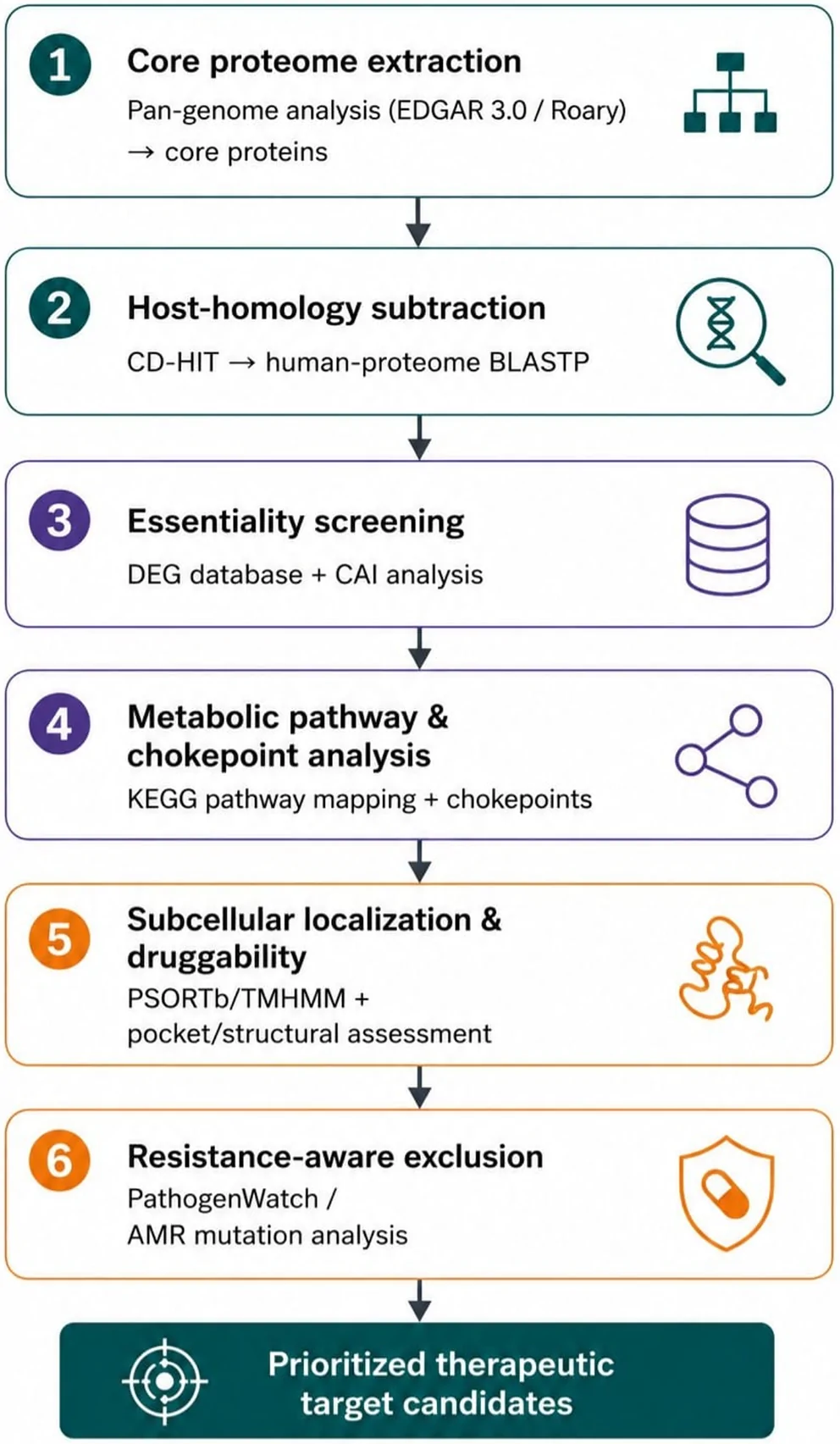
Computational proteome-to-target prioritization pipeline for *N. gonorrhoeae*. Schematic overview of an integrated six-step workflow for computational identification of candidate therapeutic targets, derived from methodologies reported across multiple gonococcal target-identification studies. The workflow sequentially applies core proteome extraction, host and microbiome homology subtraction, essentiality screening, metabolic pathway and chokepoint analysis, subcellular localization and druggability assessment, and resistance-aware exclusion.

**Table 1:** Condensed Tool Summary for the Six-Step In Silic*o* Target Fishing Pipeline

step	primary tool(s)	key parameter(s)	biological purpose	refs
1. core-genome extraction	EDGAR 3.0; roary	ANI ≥95%; core proteome threshold ≥95% strains	construct pan-genome-representative core sequence set; 12–69 strain data sets	^30,31^
2. host-homology subtraction	CD-HIT; BLASTP (*vs*. human + gut)	clustering 60–80%; E <10^–3^; identity ≤35%	eliminate host-toxic targets; reduces 12,300 → ∼101 proteins (∼13% survival)	^29,32^
3. essentiality screening	DEG v15; CAI analysis	BLASTP E ≤10^–4^; CAI threshold >0.75	confirm pathogen lethality; CAI approach bypasses annotation-dependency	^28,34,35^
4. chokepoint analysis	KAAS/KEGG	BBH-BLASTP; pathogen-*vs*.-human pathway comparison	prioritize metabolic vulnerabilities; 87.5% empirical PPV for validated targets	^36,37^
5. localization and druggability	PSORTb v3.0; TMHMM; FPocket; DrugBank	≥4 TM helices deprioritized; druggability score ≥0.5	classify subcellular compartment; assess structural pocket tractability	^28,29,38^
6. resistance-aware exclusion	Pathogenwatch AMR v2.4.9; ML framework	SNP frequency + AMR functional linkage; >20,000 WGS isolates	exclude candidates under active selective resistance pressure in clinical populations	^17,39^

### Proteome Retrieval and Core-Genome Extraction

The choice of input proteome carries more downstream consequence than any other decision in the workflow. The foundational study by Barh and Kumar used the FA 1090 reference strain exclusively,^14^ a tractable starting point that systematically misses targets diverged or absent in contemporary clinical lineages. Since *N. gonorrhoeae* harbors an open pan-genome, sampling its full clinical diversity before target identification begins is not optional.^21,29^

The methodologically sound approach is to define a core proteome: protein-coding sequences conserved across ≥95% of the input strain collection. Several computational programs can be used for core proteome definition. EDGAR 3.0 derives orthologous gene sets from precomputed pairwise alignments and remains the most widely adopted platform for this step.^30^ Omeershffudin and Kumar extracted a 12-strain gonococcal core proteome of 12,300 sequences from 84,460 publicly available *Neisseria* proteins;^29^ Qasim et al. extended this to 69 complete genomes, yielding a considerably more stringent and clinically representative core set.^21^ The size of the data set matters, with fewer than ∼10 input genomes inflating the core by retaining strain-specific genes, whereas collections exceeding 50 strains converge on the genuine conserved minimum.^21^ Roary, a rapid command-line pan-genome pipeline that clusters orthologous genes across multiple bacterial genomes, provides an efficient alternative for large-scale comparative analyses.^31^

### Host-Homology Subtraction

With the core proteome defined, the next task is to remove all proteins whose inhibition would pose collateral risk to the host or its microbiome. Three sequential BLASTP comparisons achieve this. First, intra-pathogen paralogues are resolved using CD-HIT at a 60–80% identity threshold, discarding sequences below 100 amino acids;^29,32^ in the Omeershffudin and Kumar data set, this reduced 12,300 sequences to 944 nonredundant (nonparalogous) candidates.^29^ The primary screen then compares surviving proteins against the complete *Homo sapiens* proteome; the consensus exclusion threshold across all four published gonococcal studies is *E*-value <10^–3^ with pairwise identity ≤35%.^14,21,28,29^ Only 101 proteins survived from the 944 entering this stage, corresponding to a survival rate below 10.7%, which is broadly consistent with subtractive genomics pipelines reported for other Gram-negative pathogens including *Escherichia coli*, *Klebsiella pneumoniae*, and *Pseudomonas aeruginosa*.^29^ A third comparison, against representative human gut microbiome sequences, addresses a hazard that earlier pipelines overlooked, i.e., targets sharing homology with commensal gut bacteria risk clinically meaningful dysbiosis.^21,33^ Qasim et al.^21^ explicitly incorporated this screen in their 2023 pan-genome analysis, typically eliminating a further 5–15% of candidates. Together, the three screens compressed thousands of core sequences to a tractable filtered set of 80–200 proteins.

### Essentiality Screening

In antibacterial target discovery, an “essential” gene or protein is one required for bacterial survival, replication, or maintenance under physiologically relevant conditions. Inhibiting such targets is more likely to produce bactericidal or growth-inhibitory effects, making essentiality a critical criterion in computational target prioritization. Nonhomology of the host alone is therefore insufficient. Candidate proteins must also be indispensable to the pathogen. The Database of Essential Genes (DEG v15), which integrates experimentally validated essential genes identified through genome-wide transposon mutagenesis and sequencing studies, remains the standard resource for this analysis.^34,35^ Candidate proteins are typically queried against DEG using an *E*-value threshold of ≤10^–4^.^21,29^ However, a limitation specific to *N. gonorrhoeae* must be acknowledged: gonococcal Tn-seq data sets remain considerably less comprehensive than those available for model organisms such as *E. coli* and *S. aureus*, largely because the organism’s fastidious growth requirements complicate the construction of saturating transposon insertion libraries.^14,21^ DEG screening therefore relies on cross-species homology inference, treating essentiality demonstrated in related Gram-negative organisms as surrogate evidence. This is an accepted but uncertain proxy.^14^ Tanwer et al.^28^ addressed this gap via Codon Adaptation Index (CAI) analysis, which is a sequence-intrinsic essentiality predictor entirely unaffected by annotation gaps. Genes with CAI exceeding 0.75 are inferred to encode high-priority cellular functions and applied across the full 2350-gene *N. gonorrhoeae* annotation, this criterion alone narrowed the candidate pool to 29 genes.^28^

### Metabolic Pathway and Chokepoint Analysis

Surviving proteins are assigned to KEGG metabolic modules via KEGG Automatic Annotation Server (KAAS) and compared against human pathway coverage to identify biochemical routes unique to *N. gonorrhoea*e.^36^ Within these pathways, chokepoint enzymes, which are reactions that uniquely produce or consume a specific metabolite, are prioritized because their inhibition simultaneously starves downstream pathways and causes toxic substrate accumulation.^14,37^ Approximately 87.5% of experimentally validated drug targets catalyse chokepoint reactions.^37^ Consistently identified gonococcal chokepoint pathways span lipid A biosynthesis (LpxA, LpxC, LpxD), cytoplasm-absent FAS-II fatty acid synthesis (FabI, FabH), the peptidoglycan precursor series (MurA–MurF), the diaminopimelate–lysine pathway (DapD), and folate biosynthesis (FolP, FolC).^14,21,28^

### Subcellular Localization and Druggability Assessment

PSORTb v3.0 classifies surviving candidates across all five Gram-negative compartments using five integrated detection modules, achieving precision exceeding 97% for cytoplasmic and OM categories in benchmark analyses.^38^ Transmembrane topology is assessed with TMHMM; proteins carrying four or more transmembrane helices are deprioritized for small-molecule approaches.^21,29^ Cytoplasmic proteins form the primary small-molecule target pool, whereas OM proteins (OMPs), which are surface-accessible but often poorly penetrated by antibiotics, are more suitable for antibody-based and reverse vaccinology approaches. For example, Qasim et al. identified the conserved OM-associated proteins AKP15153.1 and AKP15828.1 as potential vaccine candidates based on predicted antigenicity and surface accessibility.^21^ AKP15153.1 was defined as a protein P.IB precursor, and AKP15828.1 as a hypothetical protein. Druggability scoring evaluates whether a protein is likely to bind small therapeutic molecules with sufficient specificity and affinity. This is typically assessed by comparing candidate proteins against known drug targets in DrugBank and by identifying structurally suitable ligand-binding cavities using tools such as FPocket on experimentally derived, homology-modeled, or AlphaFold2-predicted protein structures.^28,29^

### “Resistance-Aware” Target Exclusion

Steps 1–5 of the Figure 2 pipeline represented the method limits until roughly 2020. What the earlier studies could not address is whether their shortlisted candidates were already accumulating resistance variants in circulating clinical populations. This question can now be answered through Pathogenwatch, which aggregates whole-genome sequences and computationally derived AMR profiles from over 20,000 clinical *N. gonorrhoeae* isolates across five continents.^39^ The platform’s AMR library (v2.4.9) was benchmarked against Minimum Inhibitory Concentration (MIC) data from nearly 4000 phenotypically characterized isolates.^39^

This step, Step 6 (Figure 2) cross-references each protein surviving Step 5 against two criteria, namely (1) whether its encoding gene harbors SNPs at frequencies consistent with directional selection, and (2) whether it carries any functional linkage to known resistance mechanisms. PBP2 (the *penA*-encoded transpeptidase) illustrates this filter precisely, and scores attractively on every classical subtractive genomics criterion, yet the mosaic *penA*-60.001 allele conferring ceftriaxone resistance is documented in XDR isolates across multiple continents.^39^ Resistance-aware exclusion catches this class of error and represents the single most substantive methodological advance separating post-2020 gonococcal target identification from all preceding work. The 12–30 proteins surviving this step are then advanced to the AI/ML scoring layer described below.

## Integrating AI and ML in Target Prioritization

### The Limits of Classical Subtractive Genomics as a Standalone Framework

The six-step genomics pipeline described above (Figure 2) reduces the *N. gonorrhoeae* proteome to a shortlist of 12–30 candidate proteins through a series of independent binary filters. Each step asks a yes/no question (is this protein essential? Does it share homology with the human host? Does it occupy an appropriate subcellular compartment?), but no step integrates these criteria into a continuous measure of how promising a given candidate is. The output is therefore an unranked list, not a prioritized register, and in a setting where wet-lab validation resources are finite and the therapeutic need is acute, treating all survivors as equally worthy of experimental investment is neither efficient nor scientifically defensible.^22^ Incorporating AI and ML as a ranking layer above the genomic pipeline is not an optional enhancement, but a step that converts a biological filter into a drug discovery prioritization engine (Figure 3).^17^

**Figure 3:**
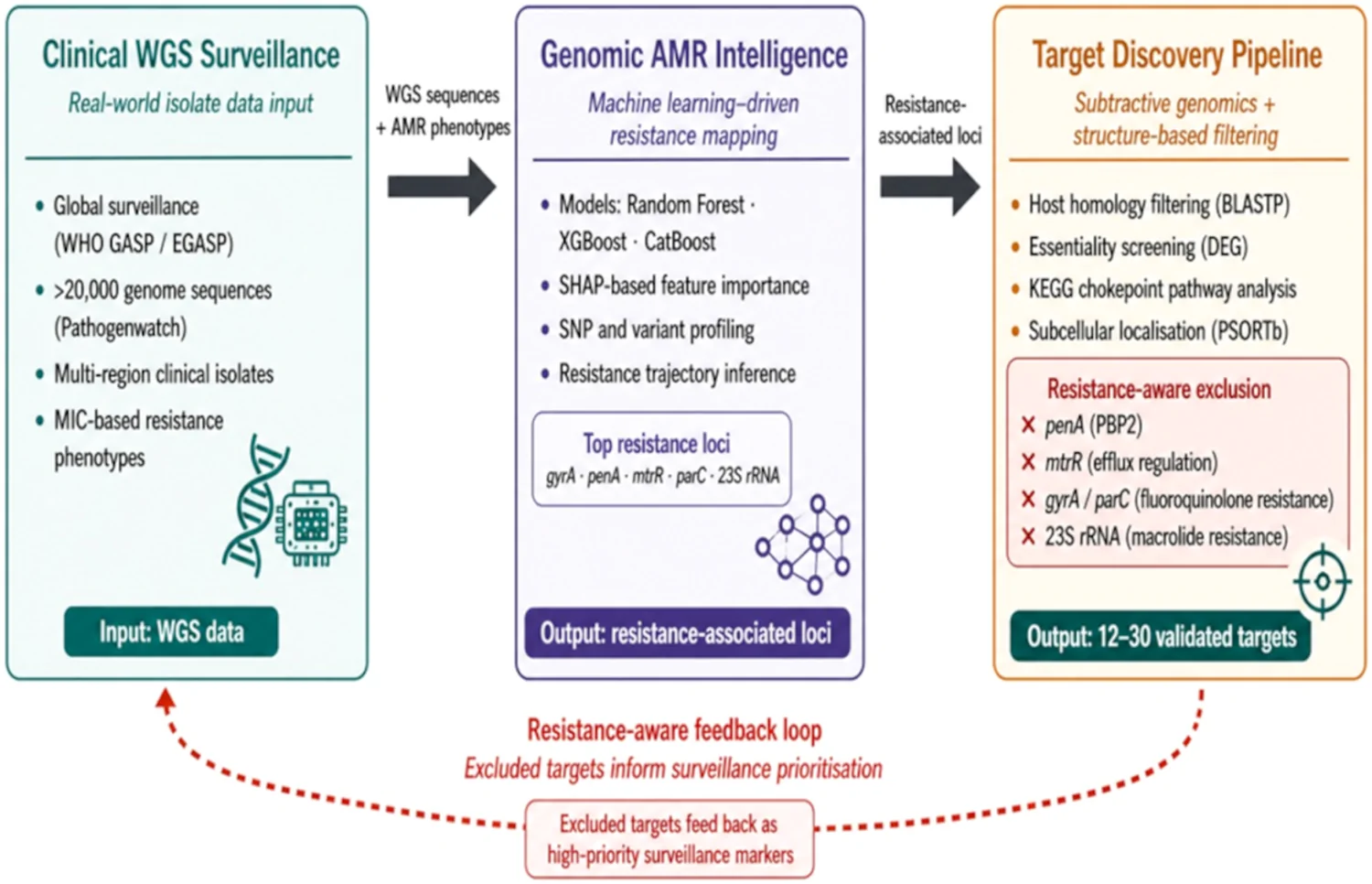
Integration of genomic AMR surveillance with resistance-aware gonococcal target discovery. A workflow illustrating how whole-genome sequencing (WGS) surveillance and ML–based resistance mapping informs subtractive genomics pipelines by excluding targets already associated with clinically established resistance determinants, thereby improving prioritization of novel therapeutic candidates in *N. gonorrhoeae*.

### ML-Based Druggability Scoring: A Priority Recommendation for Future Pipelines

Conventional druggability assessment rests on structural and pharmacological precedent, with the implicit assumption that if a protein resembles a known drug target, it is druggable. This creates a systematic blind spot for the majority of *N. gonorrhoeae* candidate proteins, which are poorly annotated, lack experimental crystal structures, and have no pharmacological literature.^22^ Under annotation-dependent approaches, these proteins will consistently score low regardless of their true biological tractability.

ML resolves this by deriving druggability probability scores from features that are computed rather than curated. Ensemble models (specifically random forest and gradient-boosted tree classifiers trained on large balanced data sets of known druggable and nondruggable proteins) encode predictions across more than 70 features per protein. These include sequence-derived descriptors (amino acid composition, predicted disorder, isoelectric point, secondary structure propensity), structural cavity properties derived from FPocket applied to AlphaFold2 models with per-residue confidence (predicted Local Distance Difference Test (pLDDT) ≥70 for backbone reliability), and protein–protein interaction network metrics, including degree and betweenness centrality.^22,40,41^ The key advantage is that none of these features requires prior experimental annotation, and poorly characterized gonococcal proteins are assessed on the same quantitative basis as well-studied targets.

It is important to state clearly that none of the four published *N. gonorrhoeae* computational target identification studies reviewed here (Barh and Kumar,^14^ Tanwer et al.,^28^ Qasim et al.^21^ or Omeershffudin and Kumar^29^) has applied ML-based druggability scoring to their candidate shortlists. This is a methodological gap that future pipeline implementations should address.

A further dimension that remains largely unexplored is microbiome-aware off-target scoring. Beaudoin et al.^42^ demonstrated that a substantial proportion of conventionally identified drug targets share meaningful sequence and structural similarity with proteins in the human gut, oral, and vaginal microbiomes, which is a promiscuity risk entirely missed by standard human-proteome BLASTP subtraction. Integrating microbiome metaproteomic similarity into ML scoring frameworks would materially improve the selectivity profile of the final target list.

### ML Models for AMR Prediction and Resistance-Informed Target Exclusion

The most practical and consequential application of ML in this pipeline is not druggability scoring but resistance prediction, explicitly, using ML-identified high-pressure genomic loci as a negative signal to exclude candidate targets whose encoding genes are already under selective pressure in clinical populations. Martin et al.^17^ constructed a sequence-based antimicrobial susceptibility testing framework trained on genome sequence and continuous MIC phenotype data from nearly 20,000 clinical *N. gonorrhoeae* isolates. Modeling continuous MIC values rather than binary classifications captures quantitative resistance trajectories that binary approaches miss entirely. Thus, a protein whose encoding locus carries even low-frequency resistance-associated variants can be flagged before phenotypic resistance is formally declared.^17^ In practice, genes ranking in the top decile of predictive importance for any modeled antibiotic class should be excluded at Step 6 of the subtractive genomics pipeline (Figure 2), regardless of how their protein product scores on structural druggability.

Kolluru et al.^43^ independently trained a CatBoost ensemble and deep neural network framework on 3786 isolates collected across 66 countries. SHapley Additive exPlanations (SHAP) interpretability analysis confirmed that canonical resistance determinants — including *gyrA* mutations for ciprofloxacin resistance and mosaic *penA* alleles for cefixime resistance — consistently ranked among the most influential predictive features, supporting that the models captured biologically meaningful resistance mechanisms rather than statistical artefacts. This SHAP-level feature attribution provides a direct, computationally grounded exclusion list for the resistance-aware filtering step.

### Deep Learning for Protein Structure Prediction

The release of AlphaFold2 in 2021 removed what had been the largest single bottleneck between computational target identification and structure-based druggability assessment, i.e., the years-long wait for experimental protein crystallization data.^40,44^ For *N. gonorrhoeae*, AlphaFold2 can provide proteome-scale structural predictions with per-residue confidence estimates, including for previously uncharacterized candidate proteins such as NGFG_RS03485,^29^ a conserved hypothetical protein prioritized through subtractive genomics despite lacking an experimentally resolved crystal structure or close structural homologue in the Protein Data Bank (PDB). Regions with pLDDT ≥70 provide reliable backbone geometry suitable for pocket detection; regions below this threshold correspond to flexible or poorly resolved segments that generate unreliable cavity predictions and should be excluded from FPocket analysis.^40,41^ Borkakoti and Thornton^41^ explicitly argued that AlphaFold2 renders previously inaccessible targets now tractable for virtual screening, fundamentally expanding the computationally druggable space.

Transformer-based protein language models, notably ESM-2 that is pre-trained across 250 million sequences, complement structural prediction by enabling functional annotation of unannotated gonococcal proteins from sequence embeddings alone.^45^ This can provide a mechanistic entry point for candidates that carry no KEGG or DEG functional annotation.

### Generative AI and De Novo Compound Design

The most forward-looking integration of AI with *N. gonorrhoeae* drug discovery was demonstrated recently by Krishnan et al.^46^ Their framework combined graph neural network–guided fragment screening of more than 45 million compounds derived from large publicly available and commercially accessible virtual chemical libraries with unconstrained de novo molecule generation using variational autoencoder and CReM genetic algorithm approaches, ultimately producing over 36 million novel candidate molecules. After filtering cytotoxicity across three human cell lines, 24 compounds progressed to experimental validation, of which seven demonstrated selective antibacterial activity against *N. gonorrhoeae*. Two lead compounds, NG1 and DN1, showed potent bactericidal activity against multidrug-resistant clinical isolates at low micromolar concentrations and significantly reduced gonococcal burden in a murine vaginal infection model.^46^ Structurally, both compounds represented previously uncharacterized small-molecule scaffolds generated through AI-guided de novo design rather than derivatives of existing gonococcal antibiotics. Mechanistic analyses suggested that their antibacterial activity involved disruption of essential bacterial membrane-associated and metabolic functions through pathways distinct from currently approved antibiotic classes, supporting their potential as first-in-class anti-gonococcal agents.

Another practical demonstration of how computational target prioritization can be translated into anti-gonococcal lead discovery was provided through the AI-driven virtual screening work performed using the Atomwise platform.^47,48^ In that study, structure-based deep learning approaches were applied to screen large chemical libraries against prioritized gonococcal protein targets involved in peptidoglycan metabolism, enabling rapid identification of candidate inhibitory compounds with predicted high-affinity binding profiles. Importantly, several computationally prioritized molecules subsequently demonstrated antibacterial activity during experimental validation, illustrating how AI-assisted target fishing can function not only as a theoretical prioritization framework but also as a practical early-stage antimicrobial discovery strategy for *N. gonorrhoeae*. This workflow complements the subtractive genomics and resistance-aware prioritization approaches discussed in the present review by providing a downstream route from computational target identification to experimentally testable chemical leads.

The connection between genomics-derived target identification and generative compound design is not incidental. The proteins identified and ranked through subtractive genomics and ML druggability scoring define the mechanistic validation benchmarks against which generative model outputs are assessed — they are the biological concepts that explain how new chemicals can be turned into useful treatments.^17,46^

## Prioritized Druggable Target Proteins — Consolidated Findings

Across the four computational studies published between 2009 and 2023, subtractive genomics and systems-level filtering have repeatedly reduced the *N. gonorrhoeae* proteome to a narrow set of candidate proteins satisfying essentiality, host nonhomology, metabolic exclusivity, appropriate subcellular localization, and resistance-trajectory safety.^14,21,28,29^ Overlap of candidates between the studies is limited, which reflects differences in strain input, DEG database version, BLASTP thresholds, and microbiome filter implementation, rather than genuine biological contradiction. To communicate translational strength clearly in this review, we apply a three-tier evidence framework: Tier 1 is cell-based antibacterial validation in *N. gonorrhoeae*; Tier 2 is target class or pathway-level clinical evidence in Gram-negative bacteria; Tier 3 is in silico prioritization without independent experimental confirmation. The ranked landscape of all candidates is illustrated in Figure 4, a consolidated target summary is shown in Table 2, and the four source studies are compared in Table 3.

**Figure 4:**
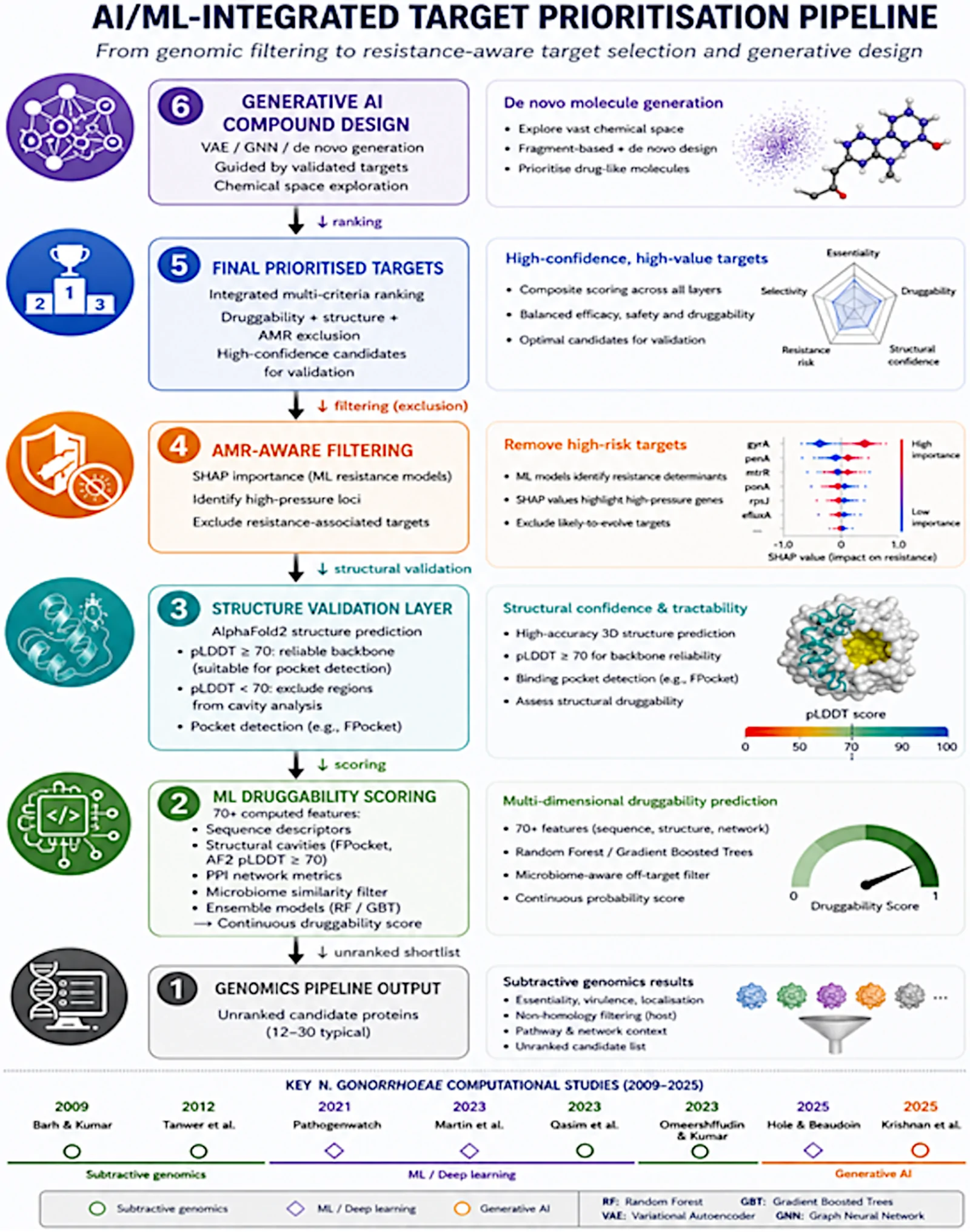
AI/ML-integrated target prioritization pipeline for *N. gonorrhoeae* drug discovery. Overview of the multi-layer computational framework integrating subtractive genomics, machine learning–based druggability prediction, AlphaFold2-enabled structural validation, resistance-aware filtering, and generative AI–driven compound design for prioritization of high-confidence anti-gonococcal therapeutic targets and novel candidate molecules. The figure summarizes the methodological progression of gonococcal computational target discovery studies from 2009–2025.

**Table 2:** Prioritized *N. gonorrhoeae* Drug Target Candidates — Consolidated Evidence

protein	gene	pathway	evidence tier	structural data	inhibitor status	refs
LpxC	*lpxC*	lipid A biosynthesis	Tier 1	AlphaFold2 model	CHIR-090; LPC-233; imidazole series (cell-based)	^14,21,52^
MurA	*murA*	peptidoglycan precursor	Tier 2	homology model	fosfomycin (class validation only)	^14,21^
FabI	*fabI*	FAS-II	Tier 2	homology model	triclosan (class); AFN-1252 (pathway tool in *Ng*)	^14,54^
MurB	*murB*	peptidoglycan precursor	Tier 2	homology model	none	^14,21^
FabH	*fabH*	FAS-II	Tier 3	homology model	class inhibitors only (thiolactomycin)	^14,55^
DapD	*dapD*	diaminopimelate/Lys	Tier 3	homology model	ZINC06311339 (predicted; unvalidated)	^28^
NGFG_RS03485	—	electron transport/redox	Tier 3	SWISS-MODEL (validated; 85.9% Ramachandran)	none — novel mechanistic class	^29,53^
AKP15153.1	*—*	OM	Tier 3 (OMP)	none	none — vaccine antigen candidate	^21^
AKP15828.1	*—*	OM	Tier 3 (OMP)	none	none — vaccine antigen candidate	^21^

**Table 3:** Comparative Summary of Published *N*. *gonorrhoeae* In Silico Target Identification Studies (2009–2023)

study	strains	key methodological addition	final targets	access
Barh and Kumar, 2009 ^14^	FA 1090	first gonococcal subtractive genomics pipeline; chokepoint analysis	106	free
Tanwer et al., 2020 ^28^	FA 1090	CAI essentiality predictor; ZINC virtual screening (DapD)	29	paid
Qasim et al., 2023 ^21^	69 (pan-genome)	gut microbiome filter; reverse vaccinology; MD simulation	12+2 OMP	free
Omeershffudin and Kumar, 2023 ^29^	12 (core proteome)	strictest published pipeline; NGFG_RS03485 identified	1	free

### Cytoplasmic Enzyme Targets

*Tier 1*. *LpxC* **—** Among all computationally proposed gonococcal targets, LpxC is the only candidate that has reached a validation level consistent with early drug discovery. Its zinc-dependent deacetylase activity occupies the first committed step of the LpxA–LpxC–LpxD lipid A pathway, and it is essential in all Gram-negative organisms and absent entirely from eukaryotic genomes.^49^ LpxC is highly conserved across Gram-negative bacteria, particularly within the catalytic zinc-binding domain required for lipid A biosynthesis, supporting its value as a broadly validated antibacterial target. However, comparative structural analyses have also identified species-specific differences within substrate-binding regions that may permit selective optimization of anti-gonococcal inhibitors while minimizing off-target activity against commensal Gram-negative organisms. Hydroxamate inhibitors CHIR-090 and PF-04753299 have demonstrated potent bactericidal activity against *N. gonorrhoeae* including XDR strain H041, with MIC values in the nanomolar-to-low micromolar range.^50^ More recent imidazole-headed chemotypes extend this activity profile.^50^ No experimental gonococcal LpxC crystal structure is deposited in the PDB, but an AlphaFold2-predicted model was used for docking studies, thereby providing a useful, though computational, structural framework.^50^

*Tier 2*. (*A*) *MurA* catalyses the first committed step of peptidoglycan precursor synthesis via an active-site cysteine intermediate. The enzyme class is clinically validated, e.g., fosfomycin exerts its antibacterial effect by covalently inhibiting this exact cysteine residue.^14^ Gonococcal-specific structural data and organism-specific inhibitor evidence are absent, keeping MurA at Tier 2 on target class validation alone.

*Tier 2*. (*B) MurB* catalyses NADPH-dependent reduction of enolpyruvyl-UDP-GlcNAc to UDP-MurNAc and appears consistently across gonococcal subtractive genomics outputs.^14,21^ Essentiality and host nonhomology are well-supported by DEG homology, but no approved inhibitor exists and no *N. gonorrhoeae*-specific experimental structure has been published.

*Tier 2*. (*C) FabI and FabH*: the bacterial FAS-II system is architecturally distinct from the mammalian FAS-I complex, conferring intrinsic pathway-level selectivity.^51,52^ FabI is inhibited by triclosan through a tight ternary NAD^+^ complex, and its homologue InhA in *M. tuberculosis* is targeted by isoniazid. These two independent precedents establish this enzyme class as pharmacologically accessible.^52^ Critically, the FabI inhibitor AFN-1252 has been used directly in *N. gonorrhoeae* to confirm that de novo FAS-II synthesis is both active and essential in this organism.^51^ FabH, which initiates fatty acid chain synthesis from acetyl-CoA and malonyl-ACP, is supported by class-level evidence only, and no gonococcal-specific inhibitor or structural data have been reported.

*Tier 3*. *DapD* sits at a bifurcation point essential for both peptidoglycan cross-link formation and de novo l-lysine production, a dual dependency absent from mammals.^28^ Tanwer et al.^28^ identified DapD through combined DEG screening and CAI analysis (CAI >0.75), then performed ZINC database virtual screening against a DapD homology model. Compound ZINC06311339 showed a zinc-chelating pharmacophore geometry complementary to the succinyl-CoA binding site. However, no biophysical or cellular confirmation of this predicted interaction has been published.

### Novel and OM Candidates

*Tier 3*. (*A*) NGFG_RS03485 was identified by Omeershffudin and Kumar^29^ as the sole cytoplasmic candidate surviving the most stringent published pipeline applied to a 12-strain *N. gonorrhoeae* core proteome. NGFG_RS03485 encodes a protein carrying a 2Fe-2S iron–sulfur cluster binding domain. BLASTP screening returned no human proteome or gut microbiome homologues. A SWISS-MODEL homology model, validated by Ramachandran analysis (85.9% residues in the favored region) and ProSA-web Z-score, supported preliminary pocket analysis and docking with cannabinoid-derived scaffolds suggested plausible binding geometries, without experimental confirmation.^29^ No approved antibiotic class currently exploits 2Fe-2S binding domain proteins in Gram-negative bacteria, making this the most mechanistically novel and chemically underexplored entry on the shortlist.

*Tier* 3. *AKP15153.1 and AKP15828.1 OMP.* These were identified by Qasim et al.^21^ through the most genetically representative analysis published, of a 69-strain core pan-genome. Both proteins are OM-localized, conserved across global clinical diversity, share no human proteome homology, and carry surface-exposed extracellular domains. Their OM location makes them poor small-molecule targets due to LOS impermeability, porin selectivity, and efflux activity,^53,54^ but they are precisely the surface-accessible, conserved, nonhost-homologous proteins that reverse vaccinology frameworks require.^21^

### Critical Appraisal

Three conclusions stand out from reading across these studies. First, LpxC is the only candidate where the computational-to-experimental trajectory has been meaningfully traversed — it alone has inhibitors demonstrating gonococcal cell-based activity at sub-microgram concentrations.^50^ Every other candidate remains anchored in computational prediction or target class analogy. Second, the low candidate overlap between studies is a reproducibility signal, not a biological one — Barh and Kumar^14^ produced 106 candidates from one strain; Omeershffudin and Kumar^29^ produced 1 from 12 strains applying a stricter pipeline to the same organism. Biology is unchanged; the methods have diverged. Third, host nonhomology is necessary but not sufficient for selectivity — Beaudoin et al.^42^ showed that pathogen drug targets can carry similarity to human microbiome proteins that standard human-proteome subtraction entirely misses. Any pipeline omitting microbiome-aware safety scoring produces an incomplete selectivity profile.

## Challenges and Critical Limitations

The pipeline reviewed above is a rigorous approach to target identification, but there are three structural failure modes, arising from database architecture, Gram-negative biology, and ML model properties, and which cannot be resolved by refinement to methodology alone.

### False Positives and the Reproducibility Gap

The reliability of any subtractive genomics pipeline is bounded by the accuracy of its input databases. DEG and KEGG are both curated from published experimental data, and both carry a systematic bias: model organisms such as *E. coli* and *B. subtilis* dominate their gene essentiality and pathway records, while *N. gonorrhoeae* is sparsely represented.^14,28^ Annotation errors in these resources propagate invisibly through the pipeline: thus, a gene misclassified as essential in DEG, or a reaction incorrectly assigned in KEGG, generates a false-positive candidate that clears every filter and reaches the final shortlist on no biological foundation.^22^

The cross-study comparison reinforces this conclusion. Placing the four gonococcal computational studies reviewed here side-by-side (Table 3) reveals candidate lists that are largely nonoverlapping, despite targeting the same organism. Tool version differences, threshold choices, strain inputs, and undetected database inconsistencies all contribute to this discrepancy. Across all four studies, most computationally proposed targets have received no experimental biochemical follow-up. The gap between a protein on a shortlist and a validated drug target is the central unresolved problem of the field.^22^

### The Gram-Negative Permeability Barrier

Correctly identifying a gonococcal cytoplasmic target and computationally docking a high-affinity compound against it is necessary but not sufficient. Whether that compound can accumulate inside the bacterium at therapeutically relevant concentrations depends on its ability to cross two sequential membrane barriers, namely the OM and the cytoplasmic membrane, a challenge that none of the four gonococcal studies reviewed here attempted to quantify.^54,55^ The Gram-negative OM is an asymmetric lipid bilayer with an outer leaflet composed predominantly of lipopolysaccharide/lipooligosaccharide.^56^ Hydrophobic compounds are largely excluded by this layer, and hydrophilic compounds must transit OM porins under size and polarity constraints that conflict directly with the Lipinski Rule of Five parameters typically used to assess drug-likeness.^54,55,57^ The eNTRy rules, which are derived from systematic physicochemical profiling of compound accumulation in Gram-negative cells, demonstrate that the parameter space for OM permeation is entirely distinct from oral bioavailability, and that conventional lipophilicity ranges actively disfavor Gram-negative penetration.^54^ A computationally valid target paired with a compound that cannot enter the organism is not a drug lead.

### Database Gaps and ML Model Extrapolation

ML druggability models are built predominantly on well-characterized mammalian and Gram-positive pathogen target data sets. When applied to the *N. gonorrhoeae* proteome, wherein a substantial proportion of proteins carry no experimentally validated functional annotation, with predictions propagated from sequence homology alone, these models extrapolate beyond their training distributions and generate false positives without any visible signal in standard performance metrics.^22^ The problem extends to AMR prediction models: the largest gonococcal training data sets contain 3786–20,000 isolates,^17,43^ a range that captures only a fraction of the species’ genotypic and phenotypic diversity globally. Reported performance metrics for gonococcal druggability prediction models should be interpreted cautiously. In most studies, model accuracy is evaluated using a subset of gonococcal data that were excluded during training and later reused for internal testing. However, these training and testing data sets often share similar annotation biases and genomic limitations, which can artificially inflate predictive performance. Importantly, prospective validation using independently collected clinical gonococcal isolates has not yet been reported for any gonococcal-specific druggability prediction framework.

## Future Directions

Four developments have the potential to substantively advance this field beyond its current state.

### AlphaFold2 Structural Proteome Mining

The AlphaFold Protein Structure Database now provides predicted three-dimensional structures for nearly all ∼2000 *N. gonorrhoeae* coding sequences.^44^ This substantially reduces the crystallographic bottleneck that historically separated computational target identification from structure-based pocket analysis. Consequently, genome-wide cavity detection across the gonococcal structural proteome is now technically feasible as a pre-investment prioritization strategy.^22,41^ However, AlphaFold2 predictions must be interpreted cautiously, particularly in regions with low pLDDT confidence scores, which frequently correspond to flexible loops, disordered segments, or poorly resolved conformations that may not reliably support docking or druggability analyses.

### Dynamic Resistance-Aware Target Exclusion

The WHO, GASP and EGASP platforms generate thousands of new gonococcal whole-genome sequences annually across five continents.^39^ If ML models were trained continuously against this incoming surveillance data and updated resistance locus rankings fed back into the Step 6 exclusion filter (Figure 4), target selection could shift from a static literature-dependent filter to a real-time clinical signal.^17,39^ This architecture remains aspirational but is technically achievable within existing surveillance infrastructure.

### Dual-Target Compound Design

Any single-target inhibitor can theoretically be defeated by a single resistance mutation. Designing compounds that simultaneously engage two cytoplasmic essential targets with no shared biochemical pathway, such as pairing FabI and LpxC, requires concurrent mutations in two independent genes, raising the genetic barrier to resistance by orders of magnitude.^21,22^ This strategy is underexplored in *N. gonorrhoeae* and becomes practical once individual target structural data are sufficiently mature for multi-target docking.

### Standardized Validation Criteria

The field currently has no community consensus on what constitutes minimum experimental evidence to advance a computational candidate to lead optimization. For example, there are no universally agreed assay panels (do we use the WHO panel, the CDCP/FDA panel, or others?), strain requirements, or structural evidence tiers.^22^ A coordinated standard, analogous to MIMARKS for sequencing studies, would directly address the reproducibility gap documented above.

## Conclusion

The continued emergence of MDR and XDR *N. gonorrhoeae* highlights the urgent need for new therapeutic strategies beyond the currently limited antibiotic options. The computational framework reviewed herein integrates core proteome analysis, host nonhomology filtering, essentiality screening, metabolic pathway assessment, resistance-aware exclusion, and AI/ML-assisted druggability prioritization to identify candidate gonococcal drug targets in a systematic manner.

Recent advances in comparative genomics, structural prediction, ML, and resistance surveillance have substantially improved the precision of computational target prioritization. However, important limitations remain, including incomplete gonococcal essentiality data sets, uncertainty in predicted structural models, and the continued need for rigorous experimental validation in clinically relevant systems. Despite these challenges, integrative in silico approaches now provide a practical and biologically informed strategy for prioritizing novel anti-gonococcal targets and may help accelerate early-stage antimicrobial discovery against this increasingly drug-resistant pathogen.

Across the reviewed studies,^14,21,28,29,50^ LpxC, MurA, FabI, DapD, and NGFG_RS03485 emerged as the most consistently prioritized gonococcal therapeutic candidates (see Table S1 for the complete cross-study candidate compilation). Among these, LpxC currently possesses the strongest experimental support, whereas NGFG_RS03485 remains a comparatively unexplored but potentially promising pathogen-specific candidate. The translational distance between these rankings and a clinical drug remains large. Not one candidate has produced a compound entering Phase I evaluation. LpxC is the furthest advanced, yet even its most promising inhibitor series has not completed validation against a representative clinical isolate panel.^50^ Computational prioritization compresses and directs the discovery process, but it cannot substitute for experimental chemistry, structural biology, or microbiology. However, the convergence of pan-genome-defined target lists, AlphaFold2 structures for every candidate, and generative AI capable of producing millions of novel compounds directly against *N. gonorrhoeae*, is arguably more formidable than anything previously reported.^21,46^ These targets, filtered through biology and resistance data, are where that generative pipeline must begin.

## Supplementary Material



## Notes

Candidate therapeutic proteins identified across four computational studies of *Neisseria* *gonorrhoeae* (Table S1) (PDF)
