# Supplementary material for: Artificial Intelligence-Integrated Proteome-Wide Target Prioritization for Drug Discovery against Multidrug-Resistant Neisseria gonorrhoeae
Source: ACS Infect Dis. 2026 Sep 9;12(10):3296–308. doi: 10.1021/acsinfecdis.6c00521 (PMC13660399; doi:10.1021/acsinfecdis.6c00521)
Supplement: Supplementary file 1 [file id6c00521_si_001.pdf]

Supplementary Information

Artificial Intelligence-Integrated Proteome-Wide Target Prioritization for Drug Discovery Against Multidrug-Resistant *Neisseria gonorrhoeae*

Ravi Kant[1], Saurabh Mazumdar[1#], Ananya Mahajan[1#], Myron Christodoulides[2*]

1. Computational Drug and Vaccine Discovery Laboratory, Faculty of Applied Sciences and Biotechnology, Shoolini University, Solan 173229, Himachal Pradesh, India
2. Neisseria Research Group, Molecular Microbiology, School of Clinical and Experimental Sciences, University of Southampton Faculty of Medicine, Southampton General Hospital, SO16 6YD Southampton, England, UK.

# These authors contributed equally to this work.

*Correspondence: Prof. Myron Christodoulides, Neisseria Research Group, Molecular Microbiology, School of Clinical and Experimental Sciences, University of Southampton Faculty of Medicine, Southampton General Hospital, SO16 6YD Southampton, England, UK. Email: M.Christodoulides@soton.ac.uk

Supplementary Table 1 - Candidate therapeutic proteins identified across four computational studies of *Neisseria gonorrhoeae*

Protein ID / Gene	Functional annotation	Barh & Kumar (2009)	Tanwer et al. (2020)	Qasim et al. (2023)	Omeershffudin & Kumar (2023)	Remarks
NGO0277	Competence lipoprotein	✓ Vaccine target	—	—	—	Surface/membrane candidate vaccine target
cysW	Sulfate transport permease protein C	✓ Vaccine target	—	—	—	Membrane transporter; strong vaccine candidate
lpxC / envA	UDP-3-O-acyl N-acetylglucosamine deacetylase	✓ Drug target	—	—	—	Lipid A biosynthesis; dual metabolic pathway involvement
rfaC	Lipopolysaccharide heptosyltransferase I	✓ Drug target	—	—	—	LPS and glycan biosynthesis
trpA	Tryptophan synthase subunit alpha	✓ Drug target	—	—	—	Amino acid biosynthesis
trpC	Indole-3-glycerol phosphate synthase	✓ Drug target	—	—	—	Two-component system-associated
trpD	Anthranilate phosphoribosyltransferase	✓ Drug target	—	—	—	Aromatic amino acid biosynthesis
YP_208704.1 (dapD)	N-succinyltransferase	✓ Drug target	✓ Lead drug target	—	—	Major docking target; lysine biosynthesis
YP_208304.1	Hypothetical essential protein	✓ Drug target	✓ Shortlisted	—	—	Essential conserved protein

AKP14928.1	Phosphoenolpyruvate-protein phosphotransferase	—	—	✓ Drug target	—	PTS system-associated
AKP14242.1	tRNA-modifying protein YgfZ	—	—	✓ Drug target	—	Translation-associated
AKP15153.1	Major outer membrane protein P.IB precursor	—	—	✓ Vaccine candidate	—	Reverse vaccinology candidate
AKP15828.1	Hypothetical outer membrane protein	—	—	✓ Vaccine candidate	—	Included in chimeric vaccine construct
NGFG_RS03485	2Fe-2S iron-sulfur cluster binding protein	—	—	—	✓ Final drug target	Passed all druggability filters
NGFG_RS11485	LpxC deacetylase	—	—	—	✓ Pre-final shortlist	LPS biosynthesis

This table summarizes candidate therapeutic proteins identified across four independent computational studies of *Neisseria gonorrhoeae* using subtractive genomics, pan-genomics, codon adaptation index (CAI)-based prioritization, and reverse vaccinology approaches.

Proteins explicitly reported in the original studies are listed along with their predicted functional annotations and occurrence across studies. (**✓**) indicates that the protein was explicitly identified or prioritized in the corresponding study, whereas (—) indicates that the protein was not reported. The table integrates drug targets, virulence-associated proteins, membrane-associated vaccine candidates, and broad-spectrum targets identified from different computational pipelines. Only proteins directly named in the source papers or associated supplementary tables were included to avoid unsupported inference.
